# Lamina‐specific population encoding of cutaneous signals in the spinal dorsal horn using multi‐electrode arrays

**DOI:** 10.1113/JP277036

**Published:** 2018-12-05

**Authors:** Charles M. Greenspon, Emma E. Battell, Ian M. Devonshire, Lucy F. Donaldson, Victoria Chapman, Gareth J. Hathway

**Affiliations:** ^1^ School of Life Sciences The University of Nottingham Nottingham NG7 2UH UK; ^2^ Bio‐Support Unit The University of Nottingham Nottingham NG7 2UH UK; ^3^ Arthritis Research UK Pain Centre The University of Nottingham Nottingham NG7 2UH UK

**Keywords:** Dorsal horn, multi‐electrode array, Somatosensory systems

## Abstract

**Key points:**

- Traditional, widely used *in vivo* electrophysiological techniques for the investigation of spinal processing of somatosensory information fail to account for the diverse functions of each lamina.
- To overcome this oversimplification, we have used multi‐electrode arrays, *in vivo*, to simultaneously record neuronal activity across all laminae of the spinal dorsal horn.
- Multi‐electrode arrays are sensitive enough to detect lamina‐ and region‐specific encoding of different subtypes of afferent fibres and to detect short‐lived changes in synaptic plasticity as measured by the application of cutaneous electrical stimulation of varying intensity and frequency.
- Differential encoding of innocuous and noxious thermal and mechanical stimuli were also detected across the laminae with the technique, as were the effects of the application of capsaicin.
- This new approach to the study of the dorsal spinal cord produces significantly more information per experiment, permitting accelerated research whilst also permitting the effects of pharmacological tools to modulate network responses.

**Abstract:**

The dorsal horn (DH) of the spinal cord is a complex laminar structure integrating peripheral signals into the central nervous system. Spinal somatosensory processing is commonly measured electrophysiologically *in vivo* by recording the activity of individual wide‐dynamic‐range neurons in the deep DH and extrapolating their behaviour to all cells in every lamina. This fails to account for the specialized processes that occur in each lamina and the considerable heterogeneity in cellular phenotype within and between laminae. Here we overcome this oversimplification by employing linear multi‐electrode arrays (MEAs) in the DH of anaesthetized rats to simultaneously measure activity across all laminae. The MEAs, comprising 16 channels, were inserted into the lumbar dorsal horn and peripheral neurons activated electrically via transcutaneous electrodes and ethologically with von Frey hairs (vFHs) or an aluminium heating block. Ascending electrical stimuli showed fibre thresholds with distinct dorsoventral innervation profiles. Wind up was observed across the DH during the C‐fibre and post‐discharge latencies following 0.5 Hz stimulation. Intrathecal application of morphine (5 ng/50 μl) significantly reduced Aδ‐ and C‐fibre‐evoked activity in deep and superficial DH. Light vFHs (≤10 g) predominantly activated intermediate and deep laminae whereas noxious vFHs (26 g) also activated the superficial laminae. Noxious heat (55°C) induced significantly greater activity in the superficial and deep laminae than the innocuous control (30°C). The application of these arrays produced the first description of the processing of innocuous and noxious stimuli throughout the intact DH.

## Key points

- Traditional, widely used *in vivo* electrophysiological techniques for the investigation of spinal processing of somatosensory information fail to account for the diverse functions of each lamina.
- To overcome this oversimplification, we have used multi‐electrode arrays, *in vivo*, to simultaneously record neuronal activity across all laminae of the spinal dorsal horn.
- Multi‐electrode arrays are sensitive enough to detect lamina‐ and region‐specific encoding of different subtypes of afferent fibres and to detect short‐lived changes in synaptic plasticity as measured by the application of cutaneous electrical stimulation of varying intensity and frequency.
- Differential encoding of innocuous and noxious thermal and mechanical stimuli were also detected across the laminae with the technique, as were the effects of the application of capsaicin.
- This new approach to the study of the dorsal spinal cord produces significantly more information per experiment, permitting accelerated research whilst also permitting the effects of pharmacological tools to modulate network responses.

## Introduction

The dorsal horn (DH) of the spinal cord is the primary site of sensory processing within the central nervous system (Basbaum *et al*. 2009; Abraira & Ginty, 2013) and is composed of a series of functionally distinct laminae, cytoarchitecturally classified (Sengul, 2015), which have been investigated with a range of techniques to help understand their functions and interactions (Yang *et al*. 2015; Ran *et al*. 2016; Hachisuka *et al*. 2016). The DH receives sensory information from cutaneous and visceral structures via primary afferent neurons and is also the target of descending projections emanating from structures within the brain, particularly the brainstem, that modulate spinal excitability (Hathway *et al*. 2009). The DH laminae (I–VI) have been broadly classified into the superficial laminae (marginal nucleus: I and substantia gelatinosa: II), which receive nociceptive information, the intermediate laminae (nucleus proprius: III–IV), which receive predominantly innocuous information, and the deep laminae (V–VI), which receive both noxious and innocuous signals (Basbaum *et al*. 2009). Crucially, as well as receiving different functional inputs, all laminae comprise different mixtures of excitatory and inhibitory interneurons, with neurons that project to supra‐spinal structures also found in several laminae (Millan, 1999). This cellular and laminar heterogeneity means that the activity of a single neuron within one region may poorly represent the activity of the larger network. Historically, *in vivo* electrophysiological recordings from the DH focused largely on lamina V wide dynamic range (WDR) neurons whilst *ex vivo* studies of synaptic physiology in the DH primarily examined the superficial laminae (Petitjean *et al*. 2015; Gutierrez‐Mecinas *et al*. 2016). Recently, intra‐vital imaging has been used to quantify population data from the DRG (Emery *et al*. 2016; Kim *et al*. 2016; Yarmolinsky *et al*. 2016) and the DH superficial laminae (Nishida *et al*. 2014; Matsumura *et al*. 2015). The predominant and segregated study of superficial and deep laminae persists despite many studies suggesting that communication between laminae, especially between the intermediate and superficial laminae, is crucial to the sensory experience and plays a major role in the development of sensory disorders (Cui *et al*. 2016; Gutierrez‐Mecinas *et al*. 2016; Yarmolinsky *et al*. 2016). Unfortunately, our understanding of the processing, modulation and relaying of information, something that is crucial to truly understand the manner in which sensation arises, is lacking due to an absence of assessment of DH function at a network or population level.

Sensory afferents are responsible for conveying information from a sensory structure to the DH and can be classified based on their physiological properties. Low threshold mechanoreceptors that transduce light tactile and vibrational signals (McGlone & Reilly, 2010) tend to terminate in the intermediate laminae and utilize predominantly (though not exclusively) Aβ‐fibres (Li *et al*. 2011; Abraira *et al*. 2017). High threshold A‐fibre nociceptor terminations are thought to be more diffuse with arbourizations present in both the superficial and the deep laminae (Woodbury *et al*. 2008; Abraira *et al*. 2017). High threshold C‐fibre nociceptors, which are better characterized than A‐fibre nociceptors, have terminals largely restricted to the superficial laminae (Olausson *et al*. 2007) but are known to synapse with deep DH neurons both directly via large dendritic trees and indirectly via interneurons (Todd, 2010; Koch *et al*. 2018). Recently, evidence has arisen suggesting that these patterns may be subject to alteration (Petitjean *et al*. 2015) in certain states, but *in vivo* techniques examining the function of this are limited in their ability and so the phenomenon is poorly understood.

The DH exhibits a somatotopic map which varies in both the anterior–posterior and the mediolateral axes (Nash *et al*. 2013). Whilst the application of intravital imaging may improve the study of inter‐segmental processing in the spinal cord, these techniques are limited by depth as only superficial neurons can be easily imaged (Pittet & Weissleder, 2011). Additionally, whilst *ex vivo* approaches are extremely useful for observation of individual neuronal properties, they come with several drawbacks such as isolation of the DH from its more widely distributed spinal networks, descending supraspinal modulatory input, and often peripheral input. Thus, an *in vivo* approach that is able to record large neuronal populations across heterogeneous structures may help to elucidate spinal mechanisms such as synaptic plasticity and network rewiring. Currently, electrophysiological studies of network activity within the DH require multiple electrodes be inserted within a small volume of the DH, resulting in potential tissue and network damage. Multi‐electrode arrays (MEAs) exploit improved manufacturing capabilities to increase the density of electrodes without increasing tissue damage (Oliveira & Dimitrov, 2008). Using MEAs in the spinal cord allows for recordings to be made from several locations simultaneously, increases the amount of data that is collected, and crucially allows comparisons between regions in the same animal.

Large arrays with low spatial resolution have been used in the spinal cord that only allow for broad comparisons between sensory (DH) and motor (ventral horn) activity (Inácio *et al*. 2016; Song & Martin, 2017). In this paper we demonstrate that 16‐electrode linear MEAs with small inter‐electrode spacing (50 μm) can be used in rats, thus significantly improving spatial resolution, to record simultaneously from all laminae of the DH. We supplied a barrage of stimuli capable of characterizing the sensory afferent fibre‐evoked activity in the DH, investigated the impact of short‐term changes in synaptic plasticity, and observed the physiological action of morphine, a commonly used analgesic, in altering DH activity. Thus MEAs can be used reliably in studies of the DH to record population activity and to facilitate the understanding of spinal networks without ignoring the functional laminar structure.

## Methods

### Ethical approval

All experiments were performed in accordance with the University of Nottingham ethical review board and were licenced by the UK Home Office (licence 40/3647 and PB3DA999F) with regards to the Animal (Scientific Procedures) Act 1986 Amendment Regulations 2012. All procedures completed were in accordance with the guidelines and requirements of *The Journal of Physiology*.

### Animals

Adult male Sprague–Dawley rats (total *n* = 18) weighing 250–300 g were sourced from Charles River UK. Animals were kept in a 12 h dark/light cycle in closed, individually ventilated cages with food and water available *ad libitum*. Cages were maintained at 21 ± 2°C and 55% humidity.

### Surgery

We adapted existing techniques utilizing linear MEAs in supraspinal structures (Kajikawa & Schroeder, 2011; Gaucher *et al*. 2012) to enable recording across the spinal cord sensory dorsal horn with sublamina resolution in some cases. Animals were anaesthetized with 4% isoflurane (Isocare, York, UK) until areflexic and transferred onto a stereotaxic frame where the anaesthetic concentration was reduced to 2.5%. A rectal probe and heating blanket (Harvard Apparatus, Holliston, MA, USA) were used to maintain core body temperature. A laminectomy was performed at the level of vertebra L1 to expose the underlying spinal cord segments L4/L5 (Gelderd & Chopin, 1977). The spinal column anterior and posterior to the laminectomy was clamped to ensure stability, and the anaesthetic concentration was reduced to 2%. The dura mater and arachnoid membrane were removed with a needle and fine forceps under a dissecting microscope to reduce resistance to electrode penetration. Following electrode placement, the anaesthetic concentration was reduced to 1.5–1.75% for the remainder of the experiment. At the end of the experiment, animals were killed by anaesthetic overdose (5% isoflurane until cessation of respiration and heart rate) with death confirmed by cervical dislocation. Throughout the experiment all ethical requirements of *The Journal* were complied with.

### Electrophysiology

**Figure 1:**
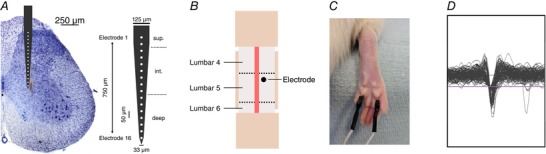
Recording multi‐unit activity across the spinal dorsal horn *A*, a 750 μm linear silicone array (16 electrodes, 50 μm spacing) was inserted vertically into L4/5 to a depth of approximately 900 μm to record from all sensory laminae. Cressyl violet stained section with electrode tract shown and overlay of electrode (to scale). Electrode represented schematically and laminar boundaries for the electrodes noted with dotted lines (superficial, intermediate and deep). *B*, following the removal of the dorsal aspect of the vertebrae and meninges the electrode was inserted into the cord where shown. *C*, stimulating electrodes were inserted under the proximal metatarsal pads so that the electrical stimulus occurred across the paw pad. *D*, example waveforms captured after cutaneous electrical stimulation using a 10% threshold (flat purple line). Window width and height is 1400 μs and 1500 μV, respectively. [Color figure can be viewed at wileyonlinelibrary.com]

**Figure 2:**
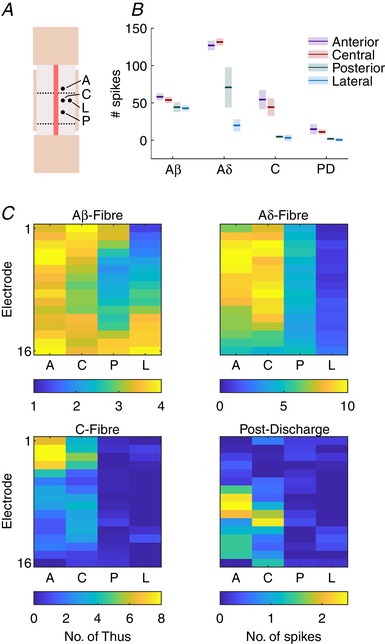
Primary afferent fibres differentially project into the spinal cord and reveal somatotopy *A*, diagrammatic representation of each location that was recorded from. The electrode was first placed in the central location and the relative locations were: 1 mm anterior, 1 mm posterior, or 0.3 mm lateral to the central location. At each location a train of 20 stimuli (5 mA, 2 ms, 0.015 Hz) was given and the evoked responses recorded (*n* = 4). *B*, summed activity of all channels at each latency period (mean ± SD) for each. *C*, heatmaps for mean evoked activity across the array (dorsoventral axis, top–bottom) at each location. Colour bar below denotes colour scale (no. of spikes) for each heatmap. [Color figure can be viewed at wileyonlinelibrary.com]

Recordings were performed using a linear silicone MEA (Fig. 1 *A*, A1x16‐5mm‐50‐177; Neuronexus, Ann Arbor, MI, USA) 15 μm thick, 33–125 μm wide, 16 electrode sites 15 μm in diameter (surface area of 177 μm^2^) with a centre to centre distance of 50 μm (total electrode range 750 μm). The array was positioned 50% along the anterior–posterior axis of the removed vertebrae (measuring from the posterior aspect of the inferior articular facet of the removed vertebrae to that of the anterior vertebrae) and 0.3 mm lateral to the central vessel (Fig. 1 *B*). To determine the reliability of this positioning method, a preliminary study (*n* = 4) was conducted by recording from three neighbouring regions (1 mm anterior or posterior to the central location, and 0.3 mm lateral to the central location; Fig. 2).

Once positioned, the electrode was advanced using a micromanipulator (IVM Single; Scientifica, Clarksburg, NJ, USA) until dimpling of the spinal cord was visible under the microscope. The array was then advanced at 20 μm s^−1^ to a depth of 1000 μm and then retracted 100 μm to reach a final depth of 900 μm to minimize distortion (Schouenborg, 1984). Stimulating electrodes were then placed into the proximal metatarsal pads of the ipsilateral hindpaw, and the average evoked local field potential (LFP) response to a train of stimuli (10 × 3 mA, 2 ms, 0.1 Hz) was recorded. The electrode was advanced or retracted so that only the deepest electrode detected a positive voltage change in response to a stimulus as opposed to the negative depolarization caused by the A‐fibre volley. Whilst low‐melt agarose has been suggested as a method of minimizing movement of the array, it prevents intrathecal drug application and so was not used in the study.

The array was attached to a headstage (HSt/16o25‐18p‐xR; 1× gain; Plexon, Dallas, TX, USA) via a custom flexible connector (Omnetics Connector Corp., Minneapolis, MN, USA). Signals were amplified by a PBX preamplifier (1000× gain; Plexon) and split into LFP (0.7–170 Hz; 5 kHz sampling frequency) and spike activity (150 Hz to 8 kHz; 40 kHz sampling frequency). Both signals were then relayed to a National Instruments (Austin, TX, USA) board on a computer where they were recorded, both with 1× gain. LFPs were continuously sampled whilst a threshold crossing of 10% below baseline was required to trigger waveform capturing in the spike channels (Fig. 1 *D*).

A pulse generator (D330; Digitimer, Welwyn Garden City, UK) was used to create a transistor–transistor logic (TTL) pulse and trigger a constant current stimulator (DS3; Digitimer) and simultaneously create an event marker on the recording software (Rasputin; Plexon). A range of stimulus intensities, durations and frequencies (*n* = 4) were delivered to the hindpaw via needle electrodes inserted into the proximal metatarsal pads to characterize the evoked spinal activity (Fig. 1 *C*). To test the effects of stimulus frequency on activity, stimuli (5 mA, 2 ms) were given at either 0.1 or 0.5 Hz (one every 10 or 2 s). A single control train (0.1 Hz) was presented followed by three wind‐up trains (0.5 Hz) with 15 min intervals between stimulus trains. To determine the effect of stimulus intensity on evoked activity, trains of twenty 2 ms pulses were presented at 0.1, 0.5, 1, 5 and 10 mA in ascending order at 0.015 Hz. A 5 min rest period was given in between trains of stimuli of different intensities. To determine the effects of stimulus duration, the same process was repeated (stimulus frequency, and interval) but with a fixed amplitude of 5 mA and stimulus durations of 0.2, 1, 2, 10 and 20 ms.

In the case of the application of morphine, a fixed stimulus (5 mA, 2 ms) was presented at 0.015 Hz. Physiological saline (50 μl) was applied and responses recorded for 30 min. The saline was then drained away under a microscope and morphine (5 ng/50 μl) was applied and the responses recorded for 90 min (*n* = 3).

To assess the effects of mechanical stimuli, von Frey hairs (1.4, 2, 6, 8, 10 and 26 g) were applied for 5 s to the centre of the plantar hindpaw, whilst for thermal stimuli an aluminium heating block driven by a pneumatic piston with a temperature of either 30 or 55°C was applied to the plantar hindpaw for 5 s (*n* = 4). In a separate group of animals (*n* = 3) a high concentration of capsaicin (50 μl of 10 mm; Sigma‐Aldrich, St Louis, MO, USA) was applied to the surface of the hindpaw and the evoked activity recorded.

At the end of some experiments, the DS3 stimulator was used to apply a 5 μA current for 2 s to produce a micro‐lesion for confirmation of depth. Animals were maintained under anaesthesia for the following 30 min to promote gliosis and lesion formation before the animal was killed via an anaesthetic overdose. Spinal cords were subsequently dissected out and placed in 4% paraformaldehyde for 3 days before being transferred into sucrose–sodium azide solution (30% sucrose, 0.5% sodium azide) for cryoprotection.

### Histology

Nissl staining was performed following fixation and slicing of the spinal cord into 50 μm sections using a cryostat (Leica 3050; Leica, Wetzlar, Germany). Sections were dehydrated and rehydrated in methanol, xylene and distilled water. Sections were placed in 1% Cresyl Violet solution for 5 min before being washed in acidic (1% acetic acid) 50% methanol and then dehydrated in methanol and cleared in xylene before coverslips were mounted with DPX (Sigma‐Aldrich). Slides were imaged using a DeltaVision microscope (GE Healthcare, Opfikon, Switzerland) and processed in GIMP for white balance.

### Data extraction and analysis

Unsorted multi‐spike activity and continuous LFPs were exported from NeuroExplorer (Plexon) to MATLAB (The MathWorks Inc., Natick, MA, USA) for processing. Custom scripts were used to add each array to a single two‐dimensional matrix upon which bulk operations could be performed. Event timings were used to extract 1.1 s epochs (100 ms before, 1 s after stimulus) for each event and channel. For multi‐unit activity (MUA) data the numbers of threshold crossings relative to the stimulus event were combined into 1 ms bins for further analysis. Two baseline calculations were performed upon the data. The average pre‐stimulus activity of the first group (subthreshold/saline) for each channel was calculated to determine baseline threshold frequency normalized to the bin size. The values were then subtracted from all bins at every stimulus to do whole channel baseline correction. In many cases subtle shifts in the baseline (pre‐stimulus) activity within trains were detected and so a second per stimulus baseline correction was performed in a similar manner. Importantly, the second baseline correction step was not performed for the datasets in which the effect of frequency was investigated. The average number of threshold crossings for each 1 ms bin at each channel was calculated for respective groups of stimuli, or per 10 min periods in the case of pharmacology studies. Latency ranges for each primary afferent fibre type with respect to the bins were calculated and the total number of threshold crossings for the latency at each channel was summed. The latencies used were based on values reported in the literature (Urch & Dickenson, 2003) that were altered slightly following preliminary experiments. Baseline/pre‐stimulus (−300 to 0 ms); Aβ (3–11 ms); Aδ (11–90 ms); C‐fibre (90–300 ms); and post‐discharge (300–800 ms). In experiments where frequency‐dependent effects were examined, the combined latency range (C‐fibre plus post‐discharge) was 90–800 ms. For mechanical and thermal stimuli, the evoked activity was summed into 100 ms bins relative to stimulus onset and for capsaicin the data were summed into 10 s bins relative to application.

In studies that investigated electrode position along the anterior–posterior/mediolateral axis, and channel‐specific responses to ranges of stimulus intensity or duration, the values were directly exported to Prism (GraphPad Software, La Jolla, CA, USA) after processing in MATLAB. In cases where region‐specific responses were investigated, channels were combined into ‘superficial’ (electrodes 1–3: 200–300 μm), ‘intermediate’ (electrodes 4–9: 350–600 μm) and ‘deep’ (electrodes 10–16: 650–900 μm; Fig. 1 *A*) subsets before values were exported to Prism.

LFPs were also examined offline to determine if any electrode drift had occurred during the experiment. Individual channels had a high‐pass filter (stop band = 0.01 Hz; pass band = 0.5 Hz) and then a notch filter (centre = 50, *Q* = 100, order = 6) applied. Whole channel baseline corrections were performed based on mean baseline voltage for the first 20 stimuli (300 ms before stimulus). Groups of LFPs were then averaged and examined to assess depth based on profile for each group. We examined the evoked LFP activity in terms of absolute amplitude (root mean squared) and time‐frequency analysis (fast Fourier transformation) but found no discernible features that gave significant insight into spinal processing (data not shown).

For figures where the MUA is presented following electrical stimulation as a heatmap with per channel activity over time, the stimulus artefact has been removed by setting all values at 0–2 ms to be equal to 0. This does not affect measurements of Aβ‐fibre activity as that is outside the 3–11 ms range used.

### Statistics

Group size was determined based on power calculations following pilot studies. Unless stated otherwise all statistical tests were parametric. Amplitude/duration data were analysed using two‐way repeated measured ANOVAs with *post hoc* Tukey's multiple comparison or Dunnett's *post hoc* test as appropriate (comparing between subsequent stimuli *vs*. comparing to baseline/subthreshold/reference location). In the case of C‐fibre and post‐discharge latencies where a depth‐specific profile was evident, datasets that did not significantly differ from baseline were excluded when testing for spatial variation. As the lowest stimulus duration used caused activation of A‐fibres, the subthreshold values from the amplitude study were used as the reference for unevoked activity. Area under the curve followed by Student's *t* test was used to determine the effects of stimulus frequency on activity. In the analysis of the effects of morphine two‐way repeated measured ANOVA was used with Dunnett's multiple comparison tests (MCT). Data were normalized to the mean evoked activity with respect to the latency period and region.

Mechanical stimulation data were assessed by comparing activity at each time point against baseline to determine how long the stimulus evoked activity for (2‐way RM‐ANOVA followed by Dunnett's MCT). For thermal stimuli a direct comparison between the stimuli was made for each 100 ms time point (2‐way RM‐ANOVA followed by Sidak's multiple comparison test).

Results in all cases were considered significant when *P* < 0.05. In associated graphs asterisks were used to represent *P*‐values in the following manner: ^*^ *P* < 0.05, ^**^ *P* < 0.01, ^***^ *P* < 0.001 and ^****^ *P* < 0.0001. In cases where there were too many points of significance to indicate, lines were used to denote that there is a significant difference but not to which degree it is different. *Z*‐score values were also calculated for all stimuli. The mean value and standard deviation for the baseline activity were calculated for each animal and electrode. On a per animal and electrode basis the mean value was then subtracted from each time point and the resulting value divided by the standard deviation calculated for that animal and electrode. *Z*‐scores across animals were then averaged and plotted as heatmaps.

### Data and code availability

All recorded data and custom code for processing, analysis and figure production are available upon request. Prism files with stored output data and completed statistical tests are also available.

## Results

A high‐density linear multi‐electrode array (16 electrodes with a centre‐to‐centre inter‐electrode spacing of 50 μm with a total range of 750 μm (Fig. 1*A*) was inserted into lumbar segments 4/5 of the DH (Takahashi *et al*. 2002; Fig. 1 *B*), following a pilot study of signal spread in the DH (Fig. 2), with the most superficial electrode in lamina I and the deepest in lamina V whilst the corresponding hindpaw was stimulated (Fig. 1 *C*).

### Thresholding and dorsoventral patterning of fibre‐evoked activity

**Figure 3:**
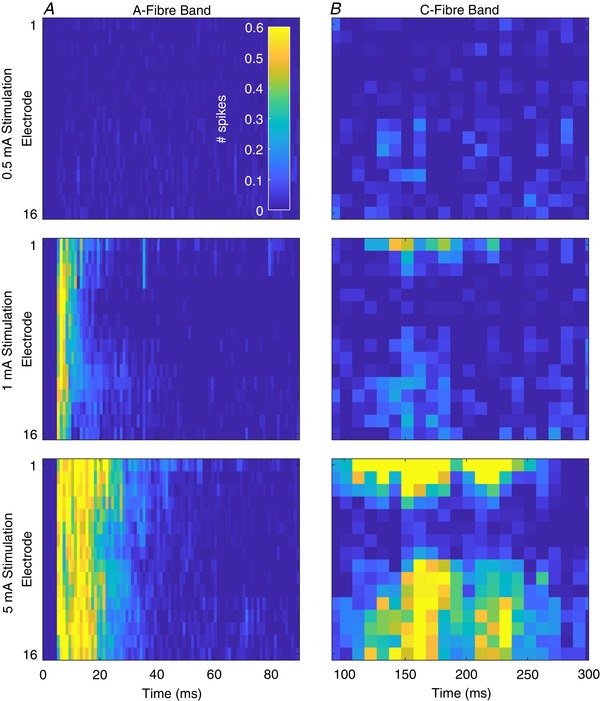
Stimulus amplitude‐dependent changes in evoked activity across the dorsal horn *A*, multi‐spike activity (MSP) in the A‐fibre band (0–90 ms after stimulus, 1 ms resolution) at each channel (dorsoventral axis of dorsal horn, top–bottom) in response to 0.5 mA (top), 1 mA (middle) and 5 mA (bottom) electrical stimulation for 2 ms. *B*, corresponding MSP activity in the C‐fibre period (90–300 ms after stimulus) in response to each stimulus (note *x*‐axis time scales). Summed activity from 10 ms (10 × 1 ms) bins are presented for each channel at each intensity. Maps represent mean response to 20 stimuli of the given intensity from 4 animals. Cell colours correspond to the legend in the top left panel. Data are from 4 animals and are the mean response to 20 stimuli of 2 ms duration at the given intensity. [Color figure can be viewed at wileyonlinelibrary.com]

**Figure 4:**
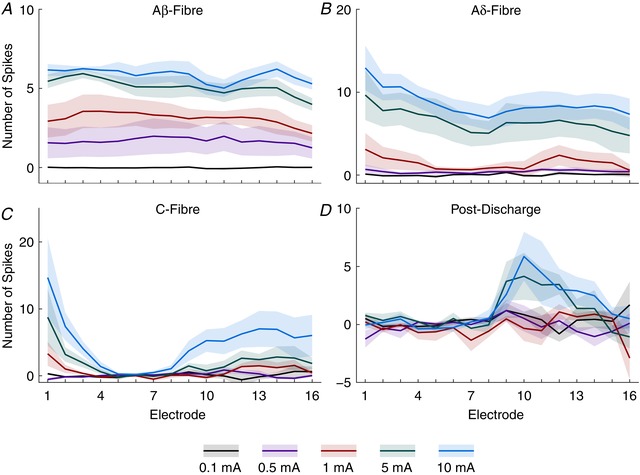
Fibre responses to electrical stimuli of increasing amplitude demonstrate specific thresholds and innervation patterns Data from Fig. 3 are shown as the summed activity over time for each fibre latency: Aβ‐ (3–11 ms), Aδ‐ (11–90 ms), C‐fibre (90–300 ms) and post‐discharge (300–800 ms) periods shown for each electrode along the dorsoventral axis and in response to each stimulus intensity. Lines represent mean value and the shaded error shows standard deviation. [Color figure can be viewed at wileyonlinelibrary.com]

Primary afferent fibres are predominantly classified based on their size and conduction velocity (Cain *et al*. 2001; McGlone & Reilly, 2010). Consequently, evoked activity can be broken down into latency periods after stimulation that are relatively specific to each fibre type (Urch & Dickenson, 2003). The biophysical properties of primary afferent fibres are such that they can be discriminated on their sensitivity to different amplitudes (rheobase) of electrical stimulation (Kiernan *et al*. 2001; Emery *et al*. 2016). As the fibres innervate each of the laminae differently it is important to record from all laminae to fully understand the network's input at rest and in response to stimulation. Consequently, square wave stimuli (2 ms width) were provided at a range of amplitudes (0.1–10 mA) to enable discrimination of fibre types based on their thresholds and latencies whilst MUA was recorded from the DH (Figs 3 and 4).

The Aβ‐fibres have the highest conduction velocity, and evoked activity in the DH to these fibres was detected between 3 and 11 ms after the stimulus (Fig. 3 *A*). The lowest stimulus amplitude to evoke a response was 0.5 mA, which only produced a response in this period (summed no. of spikes across all channels ± SD; 0.1 mA: −0.1387 ± 0.2613, 0.5 mA: 27 ± 29.31; Dunnett's MCT, 0.1 mA *vs*. 0.5 mA: *P* < 0.0001; Fig. 4 *A*). Immediately after the band of Aβ‐fibre evoked activity a second band of activity was recorded between 11 and 90 ms that occurred in response to stimuli upwards of 1 mA, which represented the Aδ‐fibres (0.1 mA: 0.6597 ± 0.4052, 1 mA: 22.73 ± 28.01; Dunnett's MCT, *P* = 0.0111; Fig. 4 *B*). The evoked activity of both A‐fibre populations did not exhibit any dorsoventral variation (2‐way RM‐ANOVA: Aβ 0.5–10 mA responses: *F*_(15,192)_ = 0.6627, *P* = 0.8188; Aδ 1–10 mA responses: *F*_(15,144)_ = 1.357, *P* = 0.1766) suggesting broad distribution of fibre input. The latencies used for the analysis differ from values found in the literature (Urch & Dickenson, 2003), which typically use latencies of 0–20 ms and 20–90 ms for Aβ‐ and Aδ‐fibres, respectively. Observation of the MUA (Fig. 3 *A*), however, shows a clear onset of the Aδ‐fibre activity at 10/11 ms after the stimulus.

The conduction velocity in C‐fibres is much slower than in A‐fibres and activity is reported to occur between 90 and 300 ms following the stimulus (Fig. 3 *B*). As they are also much thinner they exhibit higher electrical thresholds and were only activated by stimuli greater than 5 mA (0.1 mA: 1.505 ± 1.323, 5 mA: 30.53 ± 21.26; Dunnett's MCT, *P* = 0.0003; Fig. 4 *C*). The post‐discharge activity (Fig. 4 *D*; a measure of stimulus‐induced increased excitability) exhibited a U‐shaped response with moderate activity in response to 0.1 mA (5.459 ± 9.546), reduced activity in response to 0.5 and 1 mA (−1.525 ± 9.781 and −3.873 ± 11.63, respectively; Dunnett's MCT, *P* = 0.4492 and *P* = 0.2038, respectively), and increases in activity in response to 5 and 10 mA (17.66 ± 9.967 and 23.11 ± 19.03, respectively; Dunnett's MCT, *P* = 0.0580 and *P* = 0.0023, respectively). Unlike the A‐fibres, both the C‐fibre evoked and post‐discharge activity exhibited significant dorsoventral variation (2‐way RM‐ANOVA, C‐fibre 5 and 10 mA responses: *F*_(15,96)_ = 4.515, *P* < 0.0001, one‐way RM‐ANOVA; post‐discharge 10 mA response: *F*_(15,48)_ = 2.478, *P* = 0.0007).

**Figure 5:**
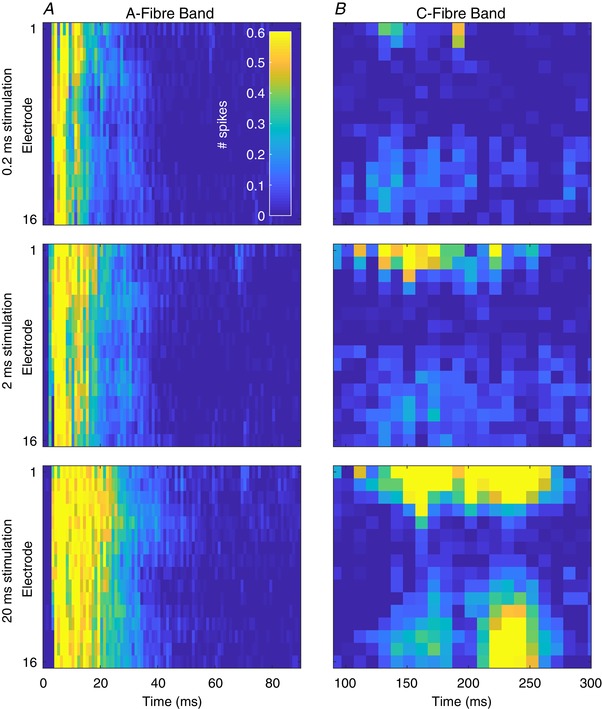
Stimulus duration dependent changes across the dorsal horn reveal fibre‐specific chronaxies *A*, multi‐spike activity (MSP) in the A‐fibre band (0–90 ms after stimulus, 1 ms resolution) at each channel (dorsoventral axis of dorsal horn, top‐bottom) in response to 1 ms (top), 2 ms (middle) and 10 ms (bottom) electrical stimulation of 5 mA. *B*, corresponding MSP activity in the C‐fibre period (90–300 ms after stimulus) in response to each stimulus (note *x*‐axis time scales). Summed activity from 10 ms (10 × 1 ms) bins are presented for each channel at each intensity. Maps represent mean response to 20 stimuli of the given intensity from 4 animals. Cell colours correspond to the legend in the top left panel. Data are from 4 animals and are the mean response to 20 stimuli of 2 ms duration at the given intensity. [Color figure can be viewed at wileyonlinelibrary.com]

**Figure 6:**
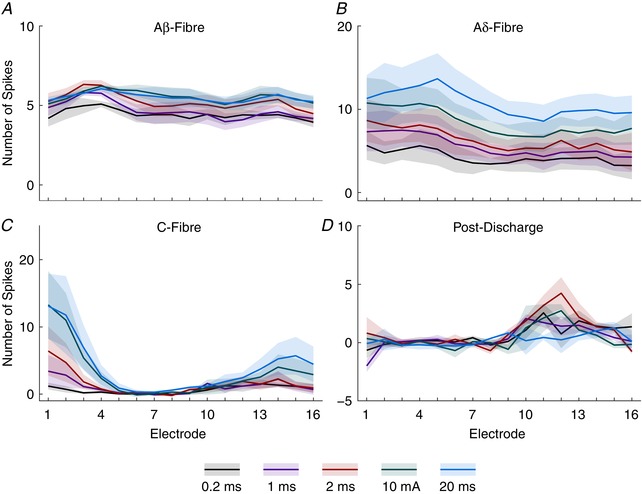
Fibre responses to electrical stimuli of increasing duration demonstrate specific thresholds and innervation patterns Data from Fig. 5 are shown as the summed activity over time for each fibre latency: Aβ‐ (3–11 ms), Aδ‐ (11–90 ms), C‐fibre (90–300 ms) and post‐discharge (300–800 ms) periods shown for each electrode along the dorsoventral axis and in response to each stimulus duration. Lines represent mean value and the shaded error shows standard deviation. [Color figure can be viewed at wileyonlinelibrary.com]

Primary afferent fibres also exhibit a relationship between the duration of the electrical stimulus and their diameter (chronaxie). The spinal implications of this, however, are poorly understood as it is not the standard paradigm to vary the duration of the electrical stimuli in spinal physiology. To complement the previous experiment, we provided trains of stimuli with a fixed amplitude (5 mA) that varied in duration (0.2–20 ms) (Figs 5 and 6).

The shortest stimulus duration (0.2 ms) given produced significant activity in the Aβ‐fibre period (71.27 ± 10.37) and increased by approximately 25% in response to the longest stimulus (88.2 ± 10.73; Dunnett's MCT, *P* < 0.0001; Fig. 6 *A*). Aδ‐fibres, similarly, evoked significant activity in response to the shortest stimulus (67.83 ± 42.37) but exhibited a much greater response range increasing up to approximately 250% in response to the longest stimulus (171.5 ± 70.29; Dunnett's MCT, *P* < 0.0001; Fig. 6 *B*). Unlike when amplitude was varied, the effect of depth on evoked activity to different durations of stimuli of both the A‐fibre subtypes exhibited significant variability (2‐way RM‐ANOVA all stimuli: Aβ: *F*_(15,240)_ = 3.19, *P* < 0.0001; Aδ: *F*_(15,240)_ = 2.082, *P* = 0.0114).

C‐fibres exhibited the largest range of activity in response to the different durations of stimuli. Minimal activity was observed in response to the 0.2 ms stimulus (10.82 ± 9.237) with activity increasing approximately 575% in response to the longest stimuli (62.31 ± 46.6; Dunnett's MCT, *P* = 0.0001; Fig. 6 *C*). Similarly to the previous study, the C‐fibre evoked activity was restricted to the superficial and deep regions of the cord (2‐way RM‐ANOVA all stimuli: *F*_(15,240)_ = 9.132, *P* < 0.0001). In this study the amount of activity evoked in response to the shortest stimulus was greater than that of the previous study (0.1 mA × 2 ms *vs*. 5 mA × 0.2 ms) resulting in a reduced range for each of the fibres in the experiment.

Finally, the post‐discharge activity (Fig. 6 *D*), interestingly, presented with an inverse profile to that in the amplitude study. The maximum activity in the post‐discharge period was in response to the 2 ms duration stimulus (14.44 ± 13.55) whilst the evoked activity in response to all other durations was significantly lower with the smallest response being to the 20 ms stimulus (4.071 ± 3.561; Dunnett's MCT *vs*. 2 ms: 20 ms, *P* = 0.0105, whilst *P* > 0.0638 for all other stimulus durations). Thus, whilst all of the fibre evoked activity produced standard growth curves, the post‐discharge activity when amplitude or duration was varied failed to do so. This suggests that post‐discharge activity may be more complex than a simple response to super‐threshold stimuli.

### Frequency‐dependent activity of DH laminae

A well‐established feature of WDR neurons in the deeper laminae is that they exhibit increased responses to high frequency stimuli (≥0.5 Hz) that can outlast the period of stimulation – a phenomenon known as wind‐up (D'Mello & Dickenson, 2008) thought to be a form of synaptic plasticity. This change in the behaviour of spinal neurons is believed to underpin aspects of central sensitization (a crucial aspect of chronic pain) as it is a clear indicator of an increase in excitability in the spinal cord that has been shown to be modulated by several drugs and within chronic pain models (D'Mello & Dickenson, 2008). Wind‐up of neurons has been shown to occur in the deep DH and to a lesser extent in the superficial DH (Seagrove *et al*. 2004; Hachisuka *et al*. 2016, 2018).

**Figure 7:**
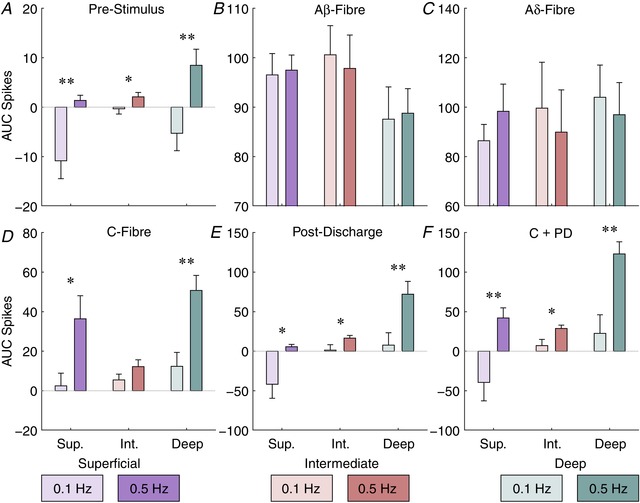
Frequency‐dependent increases in activity are restricted in temporal and spatial dimensions The total amount of activity in each latency period was measured for each stimulus in the train and the area under the curve (AUC) calculated for each stimulus frequency (0.1 or 0.5 Hz) and region. Latencies used are: pre‐stimulus, −100 to 0 ms; Aβ, 3–11 ms; Aδ, 11–90 ms; C‐fibre, 90–300 ms; post‐discharge, 300–800 ms; and the combined C‐fibre and post‐discharge (C + PD) period, 90–800 ms. Bars denote mean values whilst error bars show standard deviation. Data are from 3 animals and asterisks indicate where a paired *t* test detected a difference between trains at that latency and location. Bar colour saturation indicates stimulus frequency. [Color figure can be viewed at wileyonlinelibrary.com]

Consequently, we provided both low (0.1 Hz) and high (0.5 Hz) frequency trains of stimuli (5 mA for 2 ms; Urch & Dickenson, 2003) to determine whether the phenomenon could be recorded in deep DH neuronal networks rather than specifically in WDR neurons, and whether other network effects could be observed (Fig. 7). The high frequency train produced significant increases in evoked activity during the train in the C‐fibre latency (Fig. 7 *D*) in the superficial and deep laminae (mean area under the curve ± standard error, superficial: 0.1 Hz train: 2.479 ± 3.701, 0.5 Hz train: 36.39 ± 6.74; deep: 0.1 Hz train: 12.35 ± 4.068, 0.5 Hz train: 50.75 ± 4.387; Student's *t* test: *P* = 0.0116 and 0.003, respectively). Furthermore, in the post‐discharge period (Fig. 7 *E*) increases were seen across all regions following the 0.5 Hz train (superficial: 0.1 Hz train: −41.79 ± 10.23, 0.5 Hz train: 5.67 ± 1.703; intermediate: 0.1 Hz train: 1.38 ± 3.95, 0.5 Hz train: 16.54 ± 1.985; deep: 0.1 Hz train: 7.699 ± 9.039, 0.5 Hz train: 72.07 ± 9.364; *P* = 0.0102, 0.0266 and 0.0078, respectively). Importantly, ongoing activity outside the standard latency ranges (which we refer to as pre‐stimulus (Fig. 7 *A*) and which was counted as −100 to 0 ms) also increased between the frequency trains across the regions (superficial: 0.1 Hz train: −10.85 ± 2.091, 0.5 Hz train: 1.359 ± 0.6069; intermediate: 0.1 Hz train: −0.328 ± 0.6131, 0.5 Hz train: 2.09 ± 0.5123; deep: 0.1 Hz train: −5.285 ± 2.034, 0.5 Hz train: 8.468 ± 1.881; *P* = 0.005, 0.0389 and 0.0077, respectively). No changes in evoked activity during the trains were seen in either A‐fibre evoked latency period (Fig. 7 *B* and *C*).

Analysis of wind‐up activity, however, is conventionally done by combining the C‐fibre and post‐discharge periods (Urch & Dickenson, 2003). Performing this analysis (Fig. 7 *F*) similarly showed frequency‐dependent increases in activity across the regions (superficial: 0.1 Hz train: −39.46 ± 13.46, 0.5 Hz train: 42.06 ± 7.377; intermediate: 0.1 Hz train: 7.098 ± 4.544, 0.5 Hz train: 28.74 ± 2.446; deep: 0.1 Hz train: 22.58 ± 13.56, 0.5 Hz train: 122.8 ± 8.86; *P* = 0.006, 0.0138 and 0.0035, respectively). This suggests that wind‐up of the deep DH can be detected by the MEA, and importantly provides further evidence that wind‐up responses may occur in the superficial and intermediate DH.

### Intrathecal morphine diminishes evoked nociceptive activity

Morphine is amongst the most commonly used drugs for the treatment of pain and this is mostly due to its efficacy in inhibiting C‐fibre input, which is achieved via presynaptic opioid receptors in the DH (Heinke *et al*. 2011) and via descending control systems (Hathway *et al*. 2009). Morphine also acts upon Aδ‐fibres, though the expression of opioid receptors in this fibre type is currently contentious (Scherrer *et al*. 2009; Wang *et al*. 2010). To examine any dorsoventral axis‐dependent changes in activity of morphine we chose to topically apply it to the DH and examine changes in evoked multi‐unit activity.

**Figure 8:**
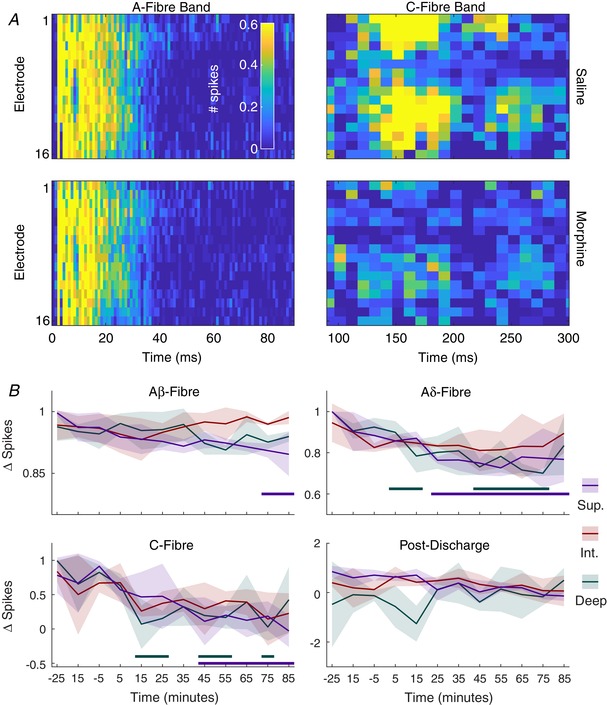
Intrathecal morphine preferentially reduces nociceptive fibre activity throughout the dorsal horn *A*, multi‐spike activity (MSP) heatmaps in the A‐fibre (0–90 ms after stimulus, 1 ms resolution) and C‐fibre (90–300 ms after stimulus, 10 ms resolution) bands at each channel (dorsoventral axis of dorsal horn, top–bottom) after 15 min of saline (−15 min relative to application of morphine) and after 15 min of morphine (5 ng/50 ul) application to the cord. Cells are 2 or 10 ms in duration and colour corresponds to the colour bar in the top left panel. *B*, data for each time point are broken into fibre latencies and regions so that specific changes may be observed. Values shown are the difference in activity between the time point and the average evoked activity during the saline period. Lines denote mean values whilst shaded areas represent standard deviation. Data are from 3 animals and thick colour coded lines below indicate where the evoked activity was significantly different from the activity in the saline period. [Color figure can be viewed at wileyonlinelibrary.com]

Following application of morphine (Fig. 8), C‐fibre evoked activity sharply dropped across the DH (2‐way RM‐ANOVA: absolute data: *F*_(12,120)_ = 6.704, *P* < 0.0001; normalized data: *F*_(12,120)_ = 5.517, *P* < 0.0001) starting at the 15 min time point and persisting for the duration of the experiment. Changes in activity were most pronounced in the superficial laminae (absolute data: 2‐way RM‐ANOVA: *F*_(12,72)_ = 8.654, *P* < 0.0001; Dunnet's MCT: *P* < 0.0093 from the 15 min time point onwards; normalized data: 2‐way RM‐ANOVA: *F*_(12,72)_ = 9.57, *P* < 0.0001; Dunnet's MCT: *P* < 0.0265 at the 15, 25, 45, 55 and 75 min time points), but significant changes were also seen in the deep laminae (Dunnet's MCT: absolute data: *P* = 0.0306 at the 85 min time point; normalized data: *P* < 0.0449 from the 45 min time point onwards). Furthermore, though no decreases were noted in the Aδ‐fibre evoked activity across the DH (Dunnet's multiple comparisons test: absolute data: *P* > 0.3428 at all time points; normalized data: *P* > 0.9902 at all time points), when examining the individual regions, significant decreases were detected in the superficial and deep DH (absolute data: 2‐way RM‐ANOVA: *F*_(12,72)_ = 9.761, *P* < 0.0001; Dunnet's MCT: superficial: *P* < 0.0296 between 15 and 75 min, deep: *P* < 0.0418 between 25 and 55 min; normalized data: 2‐way RM‐ANOVA: *F*_(12,72)_ = 11.27, *P* < 0.0001; Dunnet's MCT: superficial: *P* < 0.0479 between 15 and 75 min except for at 35 min where *P* = 0.0688; deep: *P* < 0.0321 from 25 min onwards). Finally, in the normalized data a significant decrease in Aβ‐fibre evoked activity was detected in the deep DH at the 75 and 85 min time points (2‐way RM‐ANOVA: *F*_(12,72)_ = 1.907, *P* = 0.0476; Dunnet's MCT: *P* = 0.0205 and *P* = 0.006, respectively).

### Differential stimulus intensity encoding of touch and heat across laminae

Though electrical stimulation is the best method available for activating fibres based on their biophysical properties, such as axon diameter and insulation, each fibre type exhibits functional variation (McGlone & Reilly, 2010) and this is overlooked with electrical stimulation. To assess modality‐specific encoding with the MEA we stimulated the hindpaw with mechanical and thermal stimuli.

**Figure 9:**
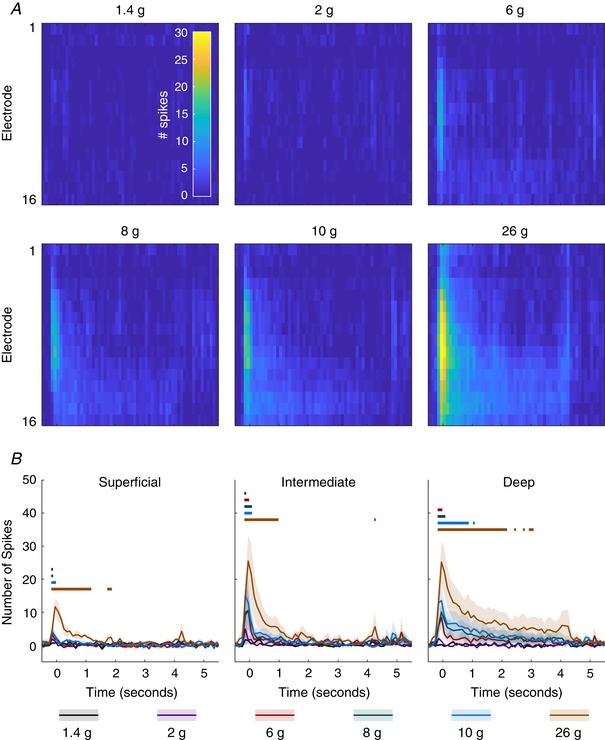
Lamina‐specific activation of graded mechanical stimuli *A*, MSP activity in response to various von Frey hairs applied to the ipsilateral hindpaw for 5 s. Cells represent summed activity in 100 ms bins and colours correspond to inset colourbar in the top left panel. *B*, activity is averaged across channels to produce activity for each region (superficial, 1:3; intermediate, 4:9; deep, 10:16). Lines indicate mean response across animals (*n* = 3) and the shaded error shows the standard deviation at each time point. Colour coded lines above indicate time points at which an ANOVA detected a significant difference between the two groups. [Color figure can be viewed at wileyonlinelibrary.com]

Encoding of the graded mechanical stimuli was evident in all laminae (2‐way RM‐ANOVA interaction statistic: *F*_(350,700)_ = 2.56, 2.711 and 3.225 for superficial, intermediate and deep, respectively, *P* < 0.0001 for all; Fig. 9). Significant activity compared to baseline was detected in the superficial DH following 6 and 8 g stimulation immediately after stimulus onset for 100 ms (Dunnet's MCT: *P* = 0.0002 and 0.0049, respectively), for 200 ms following the 10 g stimulus (*P* < 0.0110) and for 1.4 s when the 26 g hair was applied (*P* < 0.0401). In the intermediate DH, activity was first detected following 2 g stimulation for 100 ms (*P* = 0.0349), 200 ms for 6 g (*P* < 0.0001) and 300 ms for 8 and 10 g hairs (*P* < 0.0002 and *P* <0.0007, respectively). In the case of 26 g stimuli, increased activity was observed for 1.2 s (*P* < 0.0189). Finally, the deep DH showed increased responses to 6 g for 200 ms (*P* < 0.0161), 8 g for 300 ms (*P* < 0.0258), 10 g for 1.1 s (*P* < 0.0323) and 26 g for 2.4 s (*P* < 0.0044).

**Figure 10:**
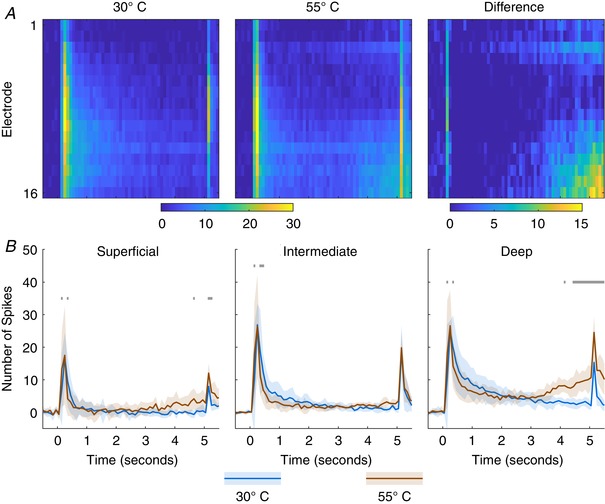
Laminae specific activation of thermal stimuli *A*, MSP activity in response to application of an innocuous (30°C) and noxious (55°C) stimulus applied to the ipsilateral hindpaw for 5 s. Cells represent summed activity in 100 ms bins and colours correspond to colourbar below. *B*, activity is averaged across channels to produce activity for each region (superficial, 1:3; intermediate, 4:9; deep, 10:16). Lines indicate mean response across animals (*n* = 3) and the shaded error shows the standard deviation at each time point. Colour coded lines above indicate time points at which an ANOVA detected a significant difference between the time point and the pre‐stimulus period. [Color figure can be viewed at wileyonlinelibrary.com]

The aluminium probe used to deliver the thermal stimuli had both mechanical and thermal components. Peaks of evoked activity were induced as a result of mechanical onset and offset whilst the effect of the probe temperature was incorporated into the response (Fig. 10). Comparing the responses at each 100 ms bin with a two‐way RM‐ANOVA and Sidak's MCT reveals two effects of the temperature on the evoked activity. During the stimulus onset significant decreases in activity were detected in all regions at the 50 (*P* < 0.0001 for all) and 250 ms (*P* < 0.0001 in the superficial and intermediate regions, *P* = 0.0072 in the deep region) time points. In the latter portion of the stimulus, as the skin is heated, significant increases in activity were detected in both the superficial (4450–4650 ms: *P* < 0.0375; 4850–5450 ms: *P* < 0.0105) and deep regions (4050 ms: *P* = 0.0254; 4350–5550 ms: *P* < 0.036). No effect of temperature was found in the in the latter half of the stimulus in the intermediate DH.

### Capsaicin application induces activity across the DH

**Figure 11:**
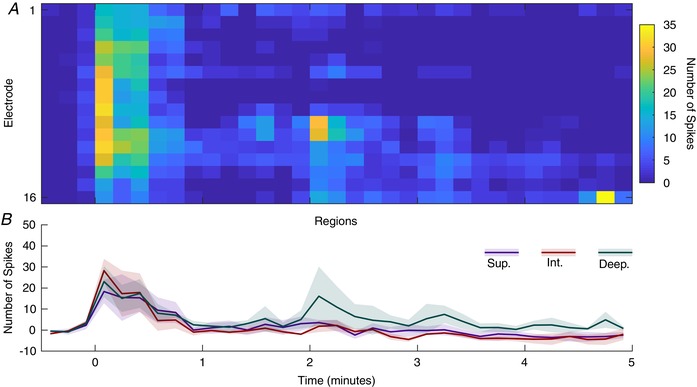
Regional and temporal effects of capsaicin application MSP activity following epicutaneous application of capsaicin (2%, 50 μl) to the plantar surface of the hindpaw. *A*, cells represent the summed activity for 100 ms and colour indicates firing rate as denoted by the colourbar on the right. *B*, corresponding regions (lines show mean values and shaded error standard deviation). Data are from 3 animals. [Color figure can be viewed at wileyonlinelibrary.com]

Capsaicin is the natural agonist of the TRPV1 receptor that is best known for its role in thermosensation and is preferentially expressed on mechanically insensitive C‐fibres (Kobayashi *et al*. 2005; Lawson *et al*. 2008). Application of capsaicin to the hindpaw (Fig. 11) induced broad activation across the DH for approximately 30 s (2‐way RM‐ANOVA: *F*_(32,192)_ = 8.218, *P* < 0.0001; Dunnet's MCT *vs*. −30 s: *P* < 0.039 for all). Though no effect was found between the regions (2‐way RM‐ANOVA: *F*_(2, 6)_ = 1.696, *P* = 0.2607), the deep region also showed a significant increase in activity at the 2 min time point (Dunnet's MCT *vs*. −30 s: *P* = 0.0246) whilst the changes in activity were not detected in the other regions.

## Discussion

Our principle aim was to determine if the linear multi‐electrode arrays are viable for recording neuronal activity from across the sensory laminae and, most importantly, if they were capable of detecting differences in responses between the laminae. Using a range of electrical stimuli, we have shown that the arrays are capable of detecting both the thresholds and dorsoventral innervation patterns of the primary afferent fibres into DH. Furthermore, we were able to detect wind‐up responses following high frequency stimuli in the deep and superficial DH despite not recording directly from wide dynamic range neurons in overcoming the documented difficulty of recording from those in the superficial laminae. We also showed that using the arrays for pharmacological studies is both practical and useful by recording robust effects of morphine that are consistent with the established literature. Finally, we demonstrate that the technique is capable of discriminating functional differences in the laminae through the application of mechanical, thermal and chemical stimuli.

### Usage of physiologically relevant stimuli

The best implementations of the linear MEA technique will be examining the spinal cords of rodents with novel sensory profiles and determining the spinal components of the phenotype. These phenotypes will be best identified and thus investigated through the use of relevant stimuli, i.e. mechanical stimuli in models that exhibit allodynia or cold stimuli in certain neuropathic models. Validation of the technique in these contexts demonstrates its putative power for the study of a range of models and mechanisms.

In the case of the mechanical stimuli, we saw a clear distinct profile in each region, with the intermediate laminae having the greatest sensitivity to low weight stimuli and the most linear encoding range with minimal thresholding, the superficial laminae only responding to the heaviest stimuli, and the deep laminae exhibiting lower thresholds than the superficial laminae but higher than the intermediate – all consistent with purported functions of the laminae (Basbaum *et al*. 2009; Dubin & Patapoutian, 2010). When examining the thermosensitive response, the building activity in the superficial and deep DH perfectly mirrors the expected response of the laminae to the heating of the paw whilst the intermediate laminae do not respond to the heat showing that the spatial restriction of input can be easily detected with the MEA.

### Wind‐up

A key element of spinal physiology research, especially with consideration to chronic pain, is the phenomenon of central sensitization, something wind‐up has been greatly implicated in (D'Mello & Dickenson, 2008). Central sensitization has been implicated as a cause of exaggerated responses to stimuli that results in chronic pain syndromes, and so the relevant mechanisms are excellent targets for pharmacological treatments. Research into wind‐up mechanisms has almost exclusively (Seagrove *et al*. 2004) focused on wind‐up of deep DH neurons as the magnitude of wind‐up is greater here and because superficial projection neurons are much smaller and thus more difficult to record from. Alternatively, optical techniques may be used, but this limits observation of neurons below the superficial laminae (Hachisuka *et al*. 2018). Crucially, the projections of the deep and superficial projection neurons differ as the superficial neurons uniquely synapse onto neurons in the parabrachial nucleus, an area which has been implicated in neuroendocrine and emotional responses to noxious stimulation (Gauriau & Bernard, 2002). In our study we found a clear wind‐up response in both the deep and superficial DH whilst the magnitude of wind‐up was markedly lower in the superficial DH (as previously reported). Interestingly the response profile of both regions was linear whereas single cell studies usually result in sigmoidal wind‐up response profiles (Fraser *et al*. 1992), possibly an integrative effect of the MUA recording approach. Consequently, the MEA approach provides a method of simultaneously recording wind‐up from both regions that will allow investigation into lamina‐specific effects of pain states and pharmacological agents. Furthermore, the MEA approach circumvents the inherent difficulty of recording from small superficial neurons and can greatly expedite the experiments.

### Post‐discharge

Post‐discharge activity has conventionally been considered to be induced as a result of C‐fibre input (Seagrove *et al*. 2004); however, it has yet to be confirmed. By providing high amplitude stimuli with short durations (5 mA for 0.2–1 ms), however, we were able to produce post‐discharge activity in the deep DH without simultaneously activating C‐fibres. Whilst in the amplitude study post‐discharge activity correlated with C‐fibre activity, it was not the case in the duration study and, in fact, post‐discharge activity does not appear to correlate with any other activity period. Furthermore, in the duration study the amount of post‐discharge activity peaked in response to 2 ms stimulation and then returned to previous levels. This suggests that post‐discharge activity may also be induced by Aδ‐fibres and that a more complex network exists to modulate post‐discharge activity.

### Morphine

The mechanisms of morphine in the DH are well documented. It thus lends itself well as a tool for confirming the viability of the MEA method for the study of DH function. Importantly, we saw the expected effect of morphine upon C‐fibre evoked activity, which showed a rapid and almost complete absence of activity following drug administration. Furthermore, we saw an interesting effect of the evoked Aδ‐fibre activity in that there was only inhibition in the superficial DH and that the inhibition only occurred much later after the morphine was administered. The latency of the effect suggests an indirect mechanism via either local spinal circuits or descending control centres. As morphine is known to only inhibit noxious sensations, the restriction of the inhibitory effects upon Aδ‐fibre evoked activity in the superficial DH is likely due to the prominence of nociceptive Aδ‐fibres in the superficial DH. Whilst Aδ‐fibres project to all regions of the DH as shown in both the amplitude and duration studies, the physiological subtypes of the Aδ‐fibres do not project across the DH (Abraira *et al*. 2017). This suggests that, given the correct stimulations and circumstances, the MEA approach may also allow for investigation of subtypes of fibres across the dorsoventral axis.

### Dorso‐ventral patterning of primary afferent fibre input

The electrical excitation thresholds for the primary afferent fibres are amongst the best established aspects of the peripheral nervous system (Urch & Dickenson, 2003). In particular Aβ‐fibres have been shown to be the quickest and easiest to activate followed by Aδ‐fibres and finally the C‐fibres. The expected response for all fibre types and activation thresholds was similar to that expected with population encoding. Importantly, however, it is well established that the size/volume of the stimulation area modulates requirements for stimulation (Rossini *et al*. 2015), and so whilst our results are consistent with those reported for the hindpaw, they may not reflect fibre thresholds for other areas such as the barrel cortex or visceral regions.

We expected an onset latency for Aδ‐fibres of approximately 20 ms based upon previously published single‐unit electrophysiological studies (Urch & Dickenson, 2003). Our measurement of this onset latency, however, conflicted with our expectations and those published studies. To ensure that the latencies we recorded were reproducible, we used animals of very similar weights so as to minimize the effect of differences in the distance evoked action potentials need to propagate from the paw to the DH as well as the conduction velocities of the fibres themselves as this changes with age and size (Rivner *et al*. 2001). In our studies we consistently recorded MUA onset at 3 ms (corresponding to the Aβ‐fibre volley of activity) and then a second burst of activity at approximately 11 ms. We conclude the latter to be the Aδ‐fibre volley, a conclusion supported by the inhibition of this activity in the superficial laminae by morphine as superficial Aδ‐fibres express opioid receptors (Wang *et al*. 2010). To accurately determine which threshold crossings result from Aβ or Aδ‐fibres based on latency alone is unachievable as there is no absolute cut‐off at which Aβ‐fibre activity stops and Aδ‐fibre activity begins. Furthermore, slight variations would be expected between cells that are ignored when observing a population. Nonetheless, our data suggest that a shorter latency for the Aδ‐fibre onset may be appropriate.

Whilst the temporally and spatially evoked activity pattern of the C‐fibres was clear, the apparent lack of variation in activity along the dorsoventral axis, evoked by either the Aβ‐ or Aδ‐fibres, was less so. This lack of patterning, however, may be due to the use of electrical stimulation. Aδ‐fibres are composed of high‐ and low‐threshold subtypes which produce different responses but have similar electrical thresholds and conduction velocities (Cain *et al*. 2001). These subtypes have different laminar innervation patterns which might be detectable if mechanical or thermal stimuli were used to activate them. With regards to the Aβ‐fibres, we expected that there would be a relatively reduced contribution of these fibres to MUA in the superficial laminae based on known terminations. Whilst innervation patterns suggest dominant activation in the intermediate laminae, it has been reported that dendrites of the neurons in the deep laminae (wide dynamic range and satellite neurons amongst others) extend well into the intermediate laminae (Todd, 2010) and so are likely to receive monosynaptic Aβ‐input. Investigations into the superficial laminae regarding neuropathic pain and allodynia have shown that the Aβ‐fibres project onto interneurons in the superficial laminae to inhibit nociception‐specific neurons and prevent the interpretation of the stimuli as being noxious (Petitjean *et al*. 2015; Cui *et al*. 2016; Arcourt *et al*. 2017), akin to the gate control theory of pain (Koch *et al*. 2018). This may explain the presence of Aβ‐fibre evoked activity in the superficial horn despite the convention that the region is specific for nociception. Due to the existence of both low‐ and high‐threshold Aβ‐ and Aδ‐fibres, an optogenetic approach to permit activation of specific fibre types or subtypes may be required to fully investigate fibre‐specific responses and network processing in the DH as the physiological stimulation used is neither specific nor temporally discrete enough.

### Mapping

Existing somatotopic maps suggest that lumbar sections 4, 5 and 6 of the spinal cord are somatotopically representative of the hindlimb and that sections 4 and 5 in particular are relevant to the hindpaw (Takahashi *et al*. 2002). As we positioned the stimulating electrodes in the plantar surface of the hindpaw, significant input signal loss was expected as the MEA moved laterally out of the plantar hindpaw area of the spinal cord and representation shifted to the dorsum of the hindpaw. The data in the pilot study reflected this extremely well with pronounced decreases in all evoked activity in the lateral region. Interestingly the activity component which decreased the least was that evoked by Aβ‐fibres potentially suggesting a role in lateral inhibition and somatotopic refinement. Activity during the Aδ‐ and C‐fibre latencies was present and consistent between the anterior and central locations (approximately L4 and L5), but much reduced in the posterior and lateral positions.

### Limitations

Whilst population encoding is considered to be very useful for measuring activity, it is not ideal for interpreting results from individual units. With this method the position of the electrode along the dorsoventral axis is imperative for recording activity from the superficial and deep laminae simultaneously, and therefore takes priority over the distance between individual electrodes and single units. If specific cell types are of interest then tetrodes would be a superior approach, though this would remove the spatial resolution. Furthermore, several electrode manufacturing companies provide ‘polytrode’ configurations (Blanche *et al*. 2005) in which one could attempt to simultaneously perform cell sorting whilst maintaining the spatial resolution we find so important in this paper. Of note, however, is that with this electrode density it would be necessary to use systems with significantly greater channel counts.

**Figure 12:**
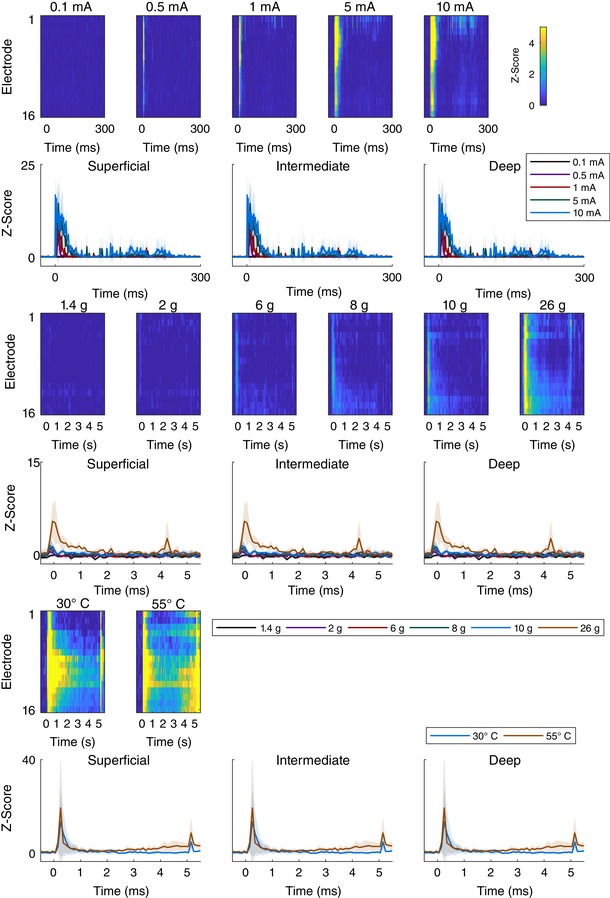
*Z*‐score values for all stimuli given The heatmaps show the corresponding *Z*‐score with respect to the channels’ pre‐stimulus mean and standard deviation computed for each animal individually and then averaged so that the heatmap could be produced. The complementary regional line graphs with standard deviation shading are shown below. The top pair of rows corresponds to electrical stimuli, the middle pair to mechanical stimuli, and the bottom pair of rows to thermal stimuli. All heatmap colours correspond to the colourbar shown in the top right whilst line graphs have their own legends to the right, below and above, respectively. The heatmap colour scale peaks at 5 standard deviations above the mean, but it is worth noting that the values far exceed that (maximum *Z*‐scores: electrical: 56; mechanical: 7; thermal: 35) and the scale has been compressed for clarity but can be seen in full on the line graphs. [Color figure can be viewed at wileyonlinelibrary.com]

Whilst the technique has the capacity to produce a tremendous amount of information about spinal processing, by using only a single shank this iteration of the approach is limited to examining how activity varies along the dorsoventral axis. As was shown in the preliminary mapping experiment, the activity varies substantially along the other axes. Though examination of adjacent regions may not be especially relevant in naive animals, evidence shows, particularly in the context of chronic pain (Henry *et al*. 2011; Kuner & Flor, 2016), that significant reorganization occurs, and this is something that has not been addressed in the spinal cord. Though we demonstrate that variation along the mediolateral and anterior–posterior axes can be detected with this technique, we posit that if non‐dorsoventral patterning were to be investigated, then a more advanced three‐dimensional array would be more appropriate. It should be cautioned, however, that this would result in significantly more neuronal displacement and damage; furthermore, an analytical method that extends beyond simple ANOVAs and Z‐scoring (Fig. 12) which is capable of computing volumetric activity akin to imaging approaches may be necessary.

### Potential complementary techniques

The usage of the linear MEA technique is not limited to naive rats. Transgenic animals allowing for optogenetic modulation of either spinal (Yang *et al*. 2015) or peripheral (Towne *et al*. 2013) neurons have enabled the activation of neural circuits to a level of precision not previously possible. The linear MEA technique may allow for real‐time *in vivo* observation of how activity propagates in the spinal DH following precise activation of these targeted cells. In particular, the assumed anatomical and functional inputs of primary afferent fibres rely on work that long predates the more accurate and precise techniques and a combination of optogenetics and the linear MEA technique may reveal much that we do not know. Furthermore, outside of optogenetics, identification of genetically discrete populations that play specific roles in spinal processing has allowed for transgenic manipulation of these circuits (Gramowski *et al*. 2004; Bourane *et al*. 2015). Behavioural and experimental measures of the induced changes have been evaluated to an extent; however, we posit that understanding the network‐level changes may be better obtained with the linear MEA technique. Crucially, this would currently require for the technique to be performed in mice or following viral transfection of rats. Though we have not attempted to do these experiments in mice, we have completed preliminary experiments in neonatal rats (which are of a comparable size) and have found no issues (data not shown).

### Conclusion

Overall, we have demonstrated that linear MEAs are a powerful tool for the investigation of the spinal cord. Despite not recording from individual neurons, our data are comparable to the majority of the published literature whilst we were able to record from multiple regions of the DH using a fraction of the number of animals, consequently taking much less time and requiring far fewer experiments. The approach also revealed several unexpected results that if coupled with other techniques may help us further elucidate the network mechanisms of the spinal dorsal horn.

## Additional information

### Competing interests

No author has any conflicting interests relating to the work in this article.

### Author contributions

GJ.H. conceived the project. C.M.G. and E.E.B. performed the experiments. C.M.G. developed the technique including surgical variations and stimulation paradigms, wrote the necessary code for data processing, analysis and figure creation, and wrote the manuscript. I.M.D. provided initial training in experimental and processing techniques to C.M.G. and assisted with generation of code used for analysis. G.J.H., L.F.D. and V.C. contributed to experimental design, interpretation of results and preparation of the manuscript. All authors had input regarding production of the manuscript. All authors have read and approved the final version of this manuscript and agree to be accountable for all aspects of the work in ensuring that questions related to the accuracy or integrity of any part of the work are appropriately investigated and resolved. All persons designated as authors qualify for authorship, and all those who qualify for authorship are listed.

### Funding

This work was supported by Arthritis Research UK [grant number 18769]; and the Biotechnology and Biological Sciences Research Council [grant numbers BB/J014508/1, BB/M008770/1]. The first grant was to the Arthritis Research UK Pain Centre (VC), and the second and third to CMG and EEB, respectively, as part of a doctoral training partnership.
